# Comparison of the adequacy of geriatric nutritional risk index with that of the mini nutritional assessment-short form and global leadership initiative on malnutrition criteria in assessing nutritional status to predict the 1-year prognosis of hospitalized Japanese older adults: a single-institutional cohort study

**DOI:** 10.1186/s12877-023-03740-5

**Published:** 2023-01-20

**Authors:** Taeko Hiraike, Chika Momoki, Daiki Habu

**Affiliations:** 1 Department of Medical Nutrition, Graduate School of Life Science, Osaka City University, 3-3-138 Sugimoto, Sumiyoshi-Ku Osaka-Shi, Osaka, 558-8585 Japan; 2 Department of Food Science and Human Nutrition, Faculty of Agriculture, Setsunan University, 45-1, Nagaotoge-Cho, Hirakata-Shi, Osaka 573-0101 Japan; 3 Department of Nutritional Medicine, Graduate School of Human Life and Ecology, Osaka Metropolitan University, 3-3-138 Sugimoto, Sumiyoshi-Ku Osaka-Shi, Osaka, 558-8585 Japan

**Keywords:** MNA^Ⓡ^-SF, GNRI, GLIM criteria, Older patients, Prognosis, Mortality, Community home support hospital

## Abstract

**Background:**

The global leadership initiative on malnutrition (GLIM) proposed the first international standards (GLIM criteria) for malnutrition diagnosis. Early screening using nutritional tools is recommended to improve the prognosis of older patients. The association between Mini Nutritional Assessment-Short Form (MNA^Ⓡ^-SF) and Geriatric Nutritional Risk Index (GNRI) and prognosis has been reported, but there is insufficient evidence to develop the GLIM criteria for older inpatients. We aimed to evaluate the MNA^Ⓡ^-SF, GNRI, and GLIM criteria to determine their contribution to the prognosis prediction of hospitalized older patients at 1 year after discharge.

**Methods:**

This study included 386 patients hospitalized between September 2014 and October 2015, and May and December 2019. After excluding 17 patients who died at the time of initial hospitalization, 23 who were lost to follow-up after 1 year, and 28 who had missing data on admission, only 318 were included in the final analysis.

The primary outcome was death within 1 year after discharge, assessed using the MNA®-SF, GNRI, and GLIM criteria, and survival analysis was conducted. Multivariate Cox proportional hazards analysis was performed to identify the nutritional assessment tools that contributed to the prognosis prediction.

**Results:**

A total of 43 patients died within 1 year. Of them, 58.1% had malnutrition and 37.2% were at risk of malnutrition, assessed using the MNA^Ⓡ^-SF; 27.9% had severely malnourished assessed using the GNRI; and 58.1% had severely malnourished assessed using the GLIM criteria. The proportions of malnourished and severely malnourished patients were significantly higher in the mortality group than in the survival group.

Multivariate Cox proportional hazards analysis showed hazard ratios of 1.06 (95% confidence interval [CI]: 0.24–4.71) for at risk and 2.17 (95% CI: 0.48–9.84) for malnutrition (MNA^Ⓡ^-SF); 5.68 (95% CI: 2.74–11.80) for moderately malnourished and 7.69 (95% CI: 3.13–18.91) for severely malnourished (GNRI); and 1.47 (95% CI: 0.48–4.50) for moderately malnourished and 2.45 (95% CI: 1.22–4.93) for severely malnourished (GLIM criteria); GNRI had the most significant contribution to prognosis prediction.

**Conclusions:**

GNRI significantly contributed to the prognosis prediction 1 year after hospital discharge of older patients.

## Background

Malnutrition in older adults leads to decreased immunity, increased susceptibility to infection [1], delayed healing of pressure ulcers and wounds [2], and decreased physical function [3], thus resulting in longer hospital stays [1], frequent readmissions [4], and increased medical costs [1].

Nutritional screening and assessment tools for older adults vary and are selected according to the type of facility, equipment, and staff capacity.

The Mini Nutritional Assessment-Short Form (MNA^Ⓡ^-SF) requires a simple interview and obtaining anthropometric measurements [5]; it does not require a blood test and is widely used in institutions and at home. The Geriatric Nutritional Risk Index (GNRI) is calculated based on the serum albumin (Alb) level and current body weight/ideal body weight ratio and is widely used as a nutritional evaluation method for hospitalized older patients [6].

In September 2018, a working group of four academic societies, European Society for Clinical Nutrition and Metabolism, American Society for Parenteral and Enteral Nutrition, Federation Latino Americana de Nutrition Parenterally Enteral, and Parenteral and Enteral Nutrition Society of Asia, developed the first international standard (global leadership initiative on malnutrition [GLIM] criteria) for malnutrition diagnosis [7]. The GLIM framework for diagnosing malnutrition is based on the phenotypic and etiological criteria. A patient is considered malnourished if he or she fulfills one of the three phenotypical criteria (weight loss, low body mass index [BMI], or reduced muscle mass) and one of the two etiological criteria (reduced food intake/assimilation or disease burden/inflammatory condition) [8]. Early screening using nutritional tools is recommended to improve the prognosis of older patients. The association between MNA^Ⓡ^-SF score and GNRI and prognosis has been reported [9–15]; however, there is insufficient evidence to establish the GLIM criteria for the severity of malnutrition in older inpatients. This prospective cohort study was the first to verify the ability of the GLIM criteria in predicting the 1-year prognosis of the oldest old inpatients with regional comprehensive functions in the rural areas of Japan, using three different purpose-built nutritional indicators (the GLIM criteria for diagnosing malnutrition, MNA^Ⓡ^-SF as a nutritional screening tool, and GNRI as a prognostic index). This study will lead to the earlier provision of nutritional interventions and lower the medical costs if appropriate nutritional tools are used in the early stage of hospitalization for older patients admitted to general hospitals in rural areas in Japan. In addition, new knowledge in the field of nutrition evaluation and nutrition-based treatment for hospitalized older patients could be created by evaluating the prognosis prediction ability of the GLIM criteria as a newly advocated nutrition evaluation method by conducting a prospective study. In the present study, the different nutritional tools contributing to the prognosis prediction of hospitalized older patients 1 year after discharge were investigated using MNA^Ⓡ^-SF, GNRI, and GLIM criteria.

## Methods

**Fig. 1:**
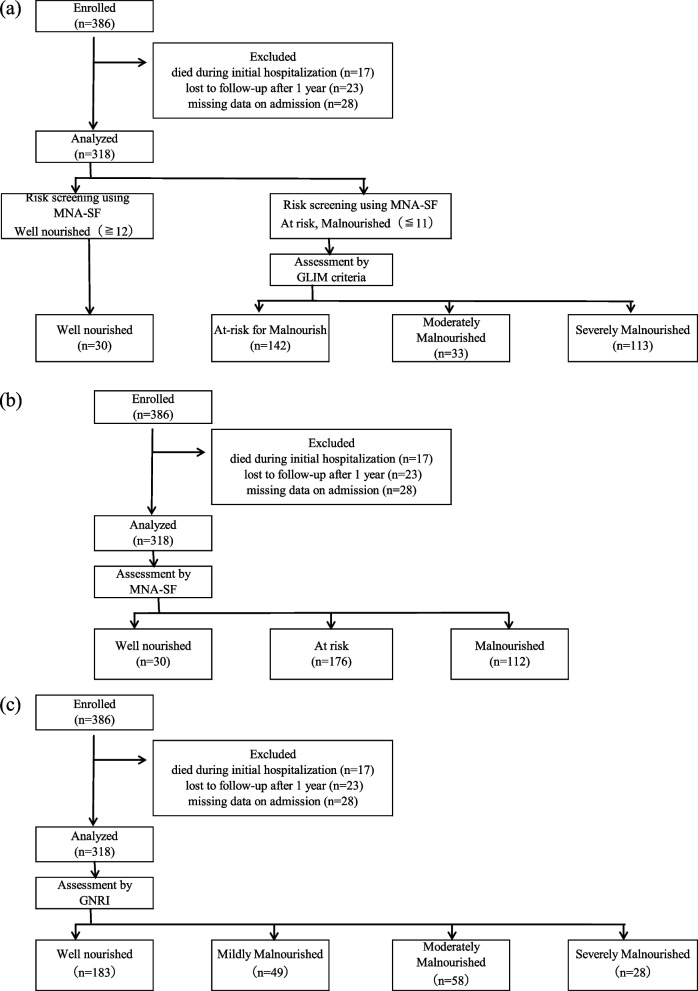
Flow diagram of the study process based on **a** GLIM criteria, **b** MNA-SF, and **c** GNRI

This single-institution prospective cohort study included 386 patients aged ≥ 65 years admitted to a home care support hospital between September 2014 and October 2015 and between May and December 2019. After excluding 17 patients who died during the initial period of hospitalization, 23 who were lost to follow-up after 1 year, and 28 who had missing data on admission, only 318 were included in the final analysis. The primary outcome was death within 1 year after discharge; based on the MNA^Ⓡ^-SF score, the patients were classified as well nourished, at risk, and malnourished. Based on the GNRI and GLIM criteria, the patients were classified as well nourished, mildly malnourished, moderately malnourished, or severely malnourished (Fig. 1).

The patients received an oral explanation about the study and signed a consent form prior to their participation. If an individual had difficulty responding, a proxy consultant was requested. This study was approved by the Ethics Committee of Okubo Hospital (no. 16).

Written informed consent was obtained from all patients or their guardians.

### Outcome

The primary outcome was the presence or absence of death within 1 year after discharge from initial admission. The patients were divided into survival and mortality groups. For patients who remained alive at the end of the study period, the end of research period was defined as the end of observation (censored).

### Assessment

Within 1 week of admission, the registered dietitian transcribed the following items from the medical record or obtained information by interviewing the patient or family members: age, sex, level of need for long-term care, prehospital situation (home [alone or otherwise], nursing home), and primary person in charge of cooking. Data of the patient’s diseases were collected from the medical records and scored using the Charlson Comorbidity Index (CCI) [16]: 0 as “low,” 1–2 as “medium,” 3–4 as “high,” and ≥ 5 as “very high.” The basic activities of daily living were assessed using the Barthel index [17].

The registered dietitian assessed the following anthropometric parameters on the day of admission. The same registered dietitian obtained the anthropometric measurements to avoid any interobserver errors. Height, weight, and triceps skinfold thickness (TSF) were measured by the same person using an adipometer (Abbott Japan Co., Ltd.). BMI was calculated on-site. The calf circumference (CC) was measured in the thickest part of the nondominant calf using a calf circumference measure (Nestle Japan Co., Ltd. Tokyo). The mean values of TSF and CC were obtained thrice.

The following laboratory parameters measured within 2 weeks of admission were obtained from the medical records: Alb, blood urea nitrogen (BUN), creatinine, red blood cell (RBC), hemoglobin (Hb), and C-reactive protein (CRP).

### Nutritional assessment

#### MNAⓇ-SF

Within one week of hospital admission, the registered dietitian interviewed the patient or a family member for evaluation. If the patient had difficulty answering the question, the situation was confirmed by a family member, a support person, a facility staff, or another primary caregiver who had knowledge on the patient’s daily dietary intake status, and the questionnaire items related to the amount of meals were evaluated. Patients who scored 12–14 points were classified as “well nourished, “8–11” points as “at risk,” and 0–7 as “malnourished.”

#### GNRI

The GNRI was calculated using the following formula:

$$\mathrm{GNRI}\:=\:\lbrack1.489\:\times\:\mathrm{Alb}(\mathrm g/\mathrm{dl})\rbrack\:+\:\lbrack41.7\:\times\:\mathrm{actual}\;\mathrm{weight}/\mathrm{ideal}\;\mathrm{body}\;\mathrm{weight}\rbrack$$

Ideal body weight was calculated using the following formula:

$$\mathrm{Ideal}\;\mathrm{body}\;\mathrm{weight}\:=\:(\mathrm{height}\:\times\:\mathrm{height})\:\times\:22$$

The calculated values were divided into the following categories: well nourished (≥ 98), mildly malnourished (92–98), moderately malnourished (82–92), and severely malnourished (< 82).

#### GLIM criteria

Based on the GLIM criteria, the severity of malnutrition was classified as moderate or severe using phenotypical grading, as described in previous studies [7, 8]. One of the three phenotypic criteria (weight loss, low BMI, or reduced muscle mass) and one of the two etiological criteria (reduced food intake/assimilation or disease burden/inflammatory condition) were used for the diagnosis and grading of malnutrition severity in all patients. Meanwhile, the MNA^Ⓡ^-SF was used for screening the risk of malnutrition. Patients with an MNA^Ⓡ^-SF score of ≥ 12 points were classified as the well-nourished group, whereas those with a score of ≤ 11 points with no actual disease and/or etiology were classified as the at-risk group. The phenotype was assessed using a BMI of < 20 kg/m^2^ (≥ 70 years < 22 kg/m^2^) and CC (< 34 cm for men and < 33 cm for women, used in the AWGS 2019 [18]). Disease burden/inflammatory condition was defined as a CRP level of > 0.5 mg/dL. Patients who met the BMI and/or CC criteria and CRP level criteria were classified as the moderately malnourished group. Patients with a BMI of < 18.5 kg/m^2^ (≥ 70 years and < 20 kg/m^2^) and/or a CC of < 31 cm (used in the MNA^Ⓡ^-SF) and met the CRP level criteria were classified as the severely malnourished group.

### Statistical analysis

The sample size was calculated using G * Power 3.1.9.2. Considering an effect size of 0.5, power of 0.8, an alpha error of 0.05, and an allocation ratio of 0.2 [19], the required sample size was 240 (group 1:200; group 2:40).

Statistical analyses were performed using IBM SPSS version 27 for Windows (IBM Japan Ltd., Tokyo, Japan) and SAS ver. 9.4 (SAS Institute Japan Co., Ltd., Tokyo, Japan). Data were expressed as mean (standard deviation [SD]), median (interquartile range), or frequency (%). The t-test or Mann–Whitney U test was used for pairwise comparisons of continuous variables between the survival and death groups. Categorical data were analyzed using the chi-square test and Fisher’s exact test.

The survival data of the groups classified according to the MNA^Ⓡ^-SF, GNRI, and GLIM criteria were analyzed using the Kaplan–Meier method. The log-rank method was used to evaluate the significant differences between the two groups.

Multivariate Cox proportional hazard analysis with mortality as an outcome was performed to adjust for the effects of confounding factors and to identify the most useful nutritional indicators for predicting patient’ prognosis. Multivariate analysis was conducted to assess the accuracy of the prognostic nutritional assessment tools, including sex, age (> 85 years), care level, prehospital situation (home [alone or other] or nursing home), CCI, MNA^Ⓡ^-SF score, GNRI, and GLIM criteria. The GNRI was divided into three categories: well nourished, mildly malnourished, moderately malnourished, and severely malnourished. The GLIM criteria were divided into three categories: well nourished and at risk for malnutrition, moderately malnourished, and severely malnourished. Considering multicollinearity, the MNA^Ⓡ^-SF, GNRI, and GLIM criteria were analyzed using separate models. In all analyses, a two-sided test was used, and a *p* value of < 0.05 was considered significant.

## Results

### Patients’ baseline characteristics

Table 1 presents the baseline characteristics of the participants. The mean age was 84.3 years (SD: 7.6), and majority of the participants were women (232, 73%).

**Table 1:** Participant's characteristics (*n* = 318)

Age (y)	84.3 (7.6)
Sex: Women	232 (73.0)
Race	
Japanese	318 (100.0)
Care level	
independent	117 (36.8)
Support care	50 (15.7)
1, 2	89 (28.0)
≧3	58 (18.2)
Missing	4
Prehospital situation	
Home (Alone)	52 (16.4)
Home (Others)	210 (66.0)
Nursing home	54 (17.0)
Missing	2
CCI (points)	
0	72 (22.6)
1–2	161 (50.6)
3–4	64 (20.1)
≧5	21 (6.6)
MNA-SF	
well nourished	30 (9.4)
At Risk	176 (55.3)
malnourished	112 (35.2)
GNRI	
well nourished	183 (57.5)
mildly malnourished	49 (15.4)
moderately malnourished	58 (18.2)
severely malnourished	28 (8.8)
GLIM	
well nourished	30 (9.4)
at-risk for malnutrition	142 (44.7)
moderately malnourished	33 (10.4)
severely malnourished	113 (35.5)
Basic ADL (points)	55 [ 25–80]
Missing	30
BMI(kg/m^2^)	
Men	22.3 ± 3.7
Women	21.9 ± 3.8
CC (cm)	
Men	31.0 ± 4.0
Women	29.1 ± 3.4
Missing	31
Albumin (g/dL)	3.6 ± 0.6
BUN (mg/dL)	21.0 ± 9.7
Creatinine (mg/dL)	0.89 ± 0.42
Red blood cell (× 10^4^/μL)	383 ± 66.7
Missing	2
Hemoglobin (g/dL)	11.8 ± 1.8
Missing	2
CRP (mg/dL)	0.68 [ 0.11–3.2]
Missing	12
Hospital stay (Days)	28 [ 17–45]

Data presented as number (percentage), mean ± SD or median [25th-75th percentile]

*CCI* Charlson comorbidity index, MNA-SF Mini nutritional assessment-Short form, *GNRI* Geriatric nutritional risk index, *GLIM* global leadership initiative on malnutrition, *DL* Activity of daily living, *BMI* Body Mass Index, CC Calf circumference, *BUN* Blood urea nitrogen, *CRP* C-reactive protein

The underlying diseases were bone and joint diseases in 135 (42.5%) patients, digestive diseases in 42 (13.2%), cerebrovascular and psychiatric diseases in 31 (9.7%), pulmonary diseases in 29 (9.1%), cardiovascular diseases in 25 (7.9%), and others in 56 (17.6%) patients.

### Survival

Table 2 shows the comparison between the two groups in terms of 1-year mortality rate. A total of 43 (13.5%) patients died within 1 year. The proportion of patients who died was relatively high in the groups that required care levels 1 and 2 (45.2%) and institutionalization (34.9%), as shown in the adjusted residual analysis. The mortality group had a significantly higher incidence of malnutrition and severely malnourished: 58.1% of the patients had malnutrition and 39.5% were at risk of malnutrition, which was assessed using the MNA^Ⓡ^-SF; 27.9% were severely malnourished, which was assessed using the GNRI; and 58.1% were severely malnourished, which was assessed using the GLIM criteria.

**Table 2:** Comparison results between the two groups for death within 1 year

	Survival	Death	*P* value
	*n* = 275	*n* = 43	
Age (y)	83.8 ± 7.7	87.3 ± 6.8	0.005
Sex: Women	205 (74.5)	27 (62.8)	0.138
Care level			
independent	109 (40.1)*	8 (19.0)*	0.003
Support care	47 (17.3)	3 (7.1)	
1, 2	70 (25.7)†	19 (45.2)†	
≧3	46 (16.9)	12 (28.6)	
Missing	3	1	
Prehospital situation			
Home (Alone)	49 (18.0)*	3 (7.0)*	0.003
Home (Others)	185 (67.8)	25 (58.1)	
Nursing home	39 (14.3)	15 (34.9)	
Missing	2		
MNA-SF	9 (7- 10)	6 (5–9)	0.001
well nourished	28 (10.2)	2 (4.7)	0.003
At Risk	160 (58.2)*	16 (37.2)*	
malnourished	87 (31.6)†	25 (58.1)†	
GNRI			
well nourished	172 (62.5)*	11 (25.6)*	< 0.001
mildly malnourished	46 (16.7)	3 (7.0)	
moderately malnourished	41 (14.9)†	17 (39.5)†	
severely malnourished	16 (5.8)‡	12 (27.9)‡	
GLIM			
well nourished	28 (10.2)	2 (4.7)	0.011
at-risk for malnutrition	130 (47.3)*	12 (27.9)*	
moderately malnourished	29 (10.5)	4 (9.3)	
severely malnourished	88 (32.0)†	25 (58.1)†	
CCI (points)			
0	69 (25.1)*	3 (7.0)*	0.003
1–2	141 (51.3)	20 (46.5)	
3–4	50 (18.2)†	14 (32.6)†	
≧5	15 (5.5)‡	6 (14.0)‡	
Basic ADL (points)	60 [ 30–85]	40 [ 15–55]	0.001
Missing	28	2	
BMI(kg/m^2^)			
Men	22.8 [ 20.7–24.8]	20.6 [16.9–22.7]	0.017
Women	21.8 [ 19.6–24.2]	21.7 [ 17.8–24.5]	0.462
TSF (mm)			
Men	6.0 [ 4.0–10.0]	4.0 [ 2.0–9.0]	0.149
Women	9.0 [ 6.0–13.0]	6.0 [ 3.5–11.0]	0.061
Missing	29	5	
CC (cm)			
Men	31.0 [ 28.7–34.2]	26.8 [25.1–31.9]	0.006
Women	29.5 [ 27.5–31.5]	27.5 [ 24.4–30.7]	0.024
Missing	28	3	
Albumin (g/dL)	3.7 ± 0.5	3.1 ± 0.6	< 0.001
BUN (mg/dL)	18.5 [ 15.0–22.9]	23.8 [ 16.8–35.2]	0.001
Creatinine (mg/dL)	0.75 [ 0.61–1.0]	0.93 [ 0.73–1.34]	0.002
Red blood cell (× 10^4^/μL)	388 ± 65	351 ± 67	0.001
Missing	2		
Hemoglobin (g/dL)	12.0 ± 1.7	10.8 ± 2.0	< 0.001
Missing	2		
CRP (mg/dL)	0.57 [ 0.10–3.10]	1.42 [ 0.22–6.31]	0.050
Missing	9	3	

Data presented as number (percentage), mean ± SD or median [25th-75th percentile]

Chi-squared and Fisher's exact tests for categorical data were performed

T test or Mann–Whitney U test for continuous data were performed

*ADL* Activity of daily living, *BMI* Body Mass Index, *TSF* Triceps skinfold thickness; CC Calf circumference, *CCI* Charlson comorbidity index, *MNA-SF* Mini nutritional assessment-Short form, *GNRI* Geriatric nutritional risk index, *GLIM* global leadership initiative on malnutrition, *BUN* Blood urea nitrogen, *CRP* C-reactive protein

^*^^, †, ‡^Significant difference by adjusted residual analysis

**Fig. 2:**
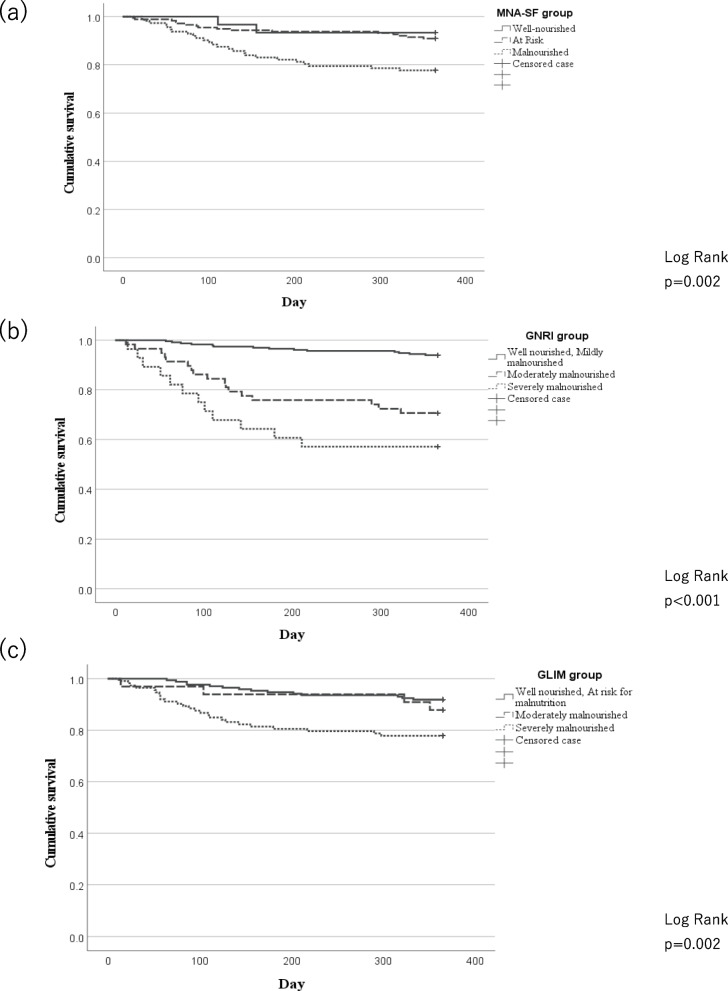
Kaplan–Meier curves for 1-year survival based on the **a** MNA-SF, **b** GNRI, and **c** GLIM criteria

The cumulative survival curves for MNA^Ⓡ^-SF, GNRI, and GLIM criteria are shown in Fig. 2. All tools showed significant results; however, the proportional hazards were not maintained for the MNA^Ⓡ^-SF and GLIM criteria.

### Outcome

In the multivariate Cox proportional hazards analysis adjusted for sex, age, prehospital situation, and CCI, the at risk of malnutrition (HR: 1.06, 95% CI: 0.24–4.71) and malnutrition groups (HR: 2.17, 95% CI: 0.48–9.84) were compared with the well-nourished group using the MNA^Ⓡ^-SF, the moderately malnourished (HR: 5.68, 95% CI: 2.74–11.80) and severely malnourished groups (HR: 7.69, 95% CI: 3.13–18.91) were compared with the well-nourished and mildly malnourished groups using the GNRI, and the moderately malnourished (HR: 1.47, 95% CI: 0.48–4.50) and severely malnourished groups (HR: 2.45, 95% CI: 1.22–4.93) were compared with the well-nourished and at risk for malnutrition groups using the GLIM criteria. Moderate and severe GNRI independently and significantly contributed to patient’s prognosis (Table 3).

**Table 3:** Multivariate Cox proportional hazard analysis with 1-year mortality

	Death *n* = 43	Crude HR	*P* value	Adjusted HR model 1	*P* value	Adjusted HR model 2	*P* value	Adjusted HR model 3	*P* value
Age: ≧85	26 (60.5)	1.49 (0.81–2.75)	0.201	1.30 (0.66–2.56)	0.449	1.30 (0.65–2.56)	0.458	1.24 (0.62–2.51)	0.542
Sex: Women	27 (62.8)	0.61 (0.33–1.13)	0.117	0.54 (0.27–1.09)	0.087	0.53 (0.26–1.09)	0.083	0.50 (0.24–1.03)	0.061
Care level									
independent	8 (19.0)								
Support care	3 (7.1)	0.89 (0.24–3.35)	0.862						
1, 2	19 (45.2)	3.46 (1.51–7.90)	0.003						
≧3	12 (28.6)	3.26 (1.33–7.99)	0.010						
Missing	1								
		Trend *p* = 0.001							
Prehospital situation									
Home (Alone)	3 (7.0)								
Home (Others)	25 (58.1)	0.18 (0.05–0.61)	0.006	0.38 (0.10–1.42)	0.149	0.36 (0.10–1.34)	0.129	0.78 (0.19–3.19)	0.734
Nursing home	15 (34.9)	0.38 (0.20–0.73)	0.003	0.53 (0.26–1.10)	0.089	0.52 (0.26–1.04)	0.066	0.91 (0.40–2.10)	0.829
		Trend *p* = 0.014		Trend *p* = 0.132		Trend *p* = 0.097		Trend *p* = 0.878	
CCI (points)									
0	3 (7.0)								
1–2	20 (46.5)	3.11 (0.93–10.47)	0.067	2.45 (0.72–8.38)	0.154	2.55 (0.74–8.76)	0.137	2.49 (0.73–8.53)	0.146
3–4	14 (32.6)	5.90 (1.70–20.54)	0.005	3.47 (0.94–12.80)	0.062	3.45 (0.94–12.70)	0.063	4.00 (1.06–15.11)	0.041
≧5	6 (14.0)	8.56 (2.14–34.22)	0.002	5.95 (1.44–24.60)	0.014	5.50 (1.31–23.18)	0.020	5.92 (1.42–24.63)	0.014
		Trend *p* < 0.001		Trend *p* = 0.008		Trend *p* = 0.014		Trend *p* = 0.006	
MNA-SF									
well nourished	2 (4.7)								
At Risk	16 (37.2)	1.39 (0.32–6.04)	0.661	1.06 (0.24–4.71)	0.941				
malnourished	25 (58.1)	3.70 (0.88–15.61)	0.075	2.17 (0.48–9.84)	0.316				
		Trend *p* = 0.002		Trend *p* = 0.036					
GNRI									
well nourished and mildly malnourished	14 (32.6)								
moderately malnourished	17 (39.5)	5.65 (2.79–11.47)	< 0.001					5.68 (2.74–11.80)	< 0.001
severely malnourished	12 (27.9)	9.57 (4.42–20.73)	< 0.001					7.69 (3.13–18.91)	< 0.001
		Trend *p* < 0.001						Trend *p* < 0.001	
GLIM									
well nourished and at-risk for malnutrition	14 (32.6)								
moderately malnourished	4 (9.3)	1.52 (0.50–4.60)	0.463			1.47 (0.48–4.50)	0.499		
severely malnourished	25 (58.1)	3.04 (1.58–5.85)	< 0.001			2.45 (1.22–4.93)	0.012		
		Trend *p* < 0.001				Trend *p* = 0.012			

Model 1 included age(≧85), sex(women), prehospital situation, CCI and MNA-SF

Model 2 included age(≧85), sex(women), prehospital situation, CCI and GNRI

Model 3 included age(≧85), sex(women),prehospital situation, CCI and GLIM

Data presented as HR (95% CI). *HR* hazard ratio, *CI* confidence interval

*CCI* Charlson comorbidity index, *MNA-SF* Mini nutritional assessment-Short form, *GNRI* Geriatric nutritional risk index, *GLIM* global leadership initiative on malnutrition

## Discussion

This cohort prospectively compared the 1-year prognosis of the oldest old patients admitted to core hospitals in rural Japan using three different purpose-built tools: MNA-SF, which has been used as a nutritional screening method for older patients; GNRI, which has been used as a nutritional prognostic indicator using objective data assessment; and GLIM criteria, which has been recently adopted as a nutritional diagnostic method. As a result, the GNRI, which is simple and does not require special inquiry or special examination, was superior in predicting the prognosis after 1 year.

The GNRI is a nutritional screening tool specific to older individuals and can be calculated based on body weight and serum Alb levels. Detailed interviews were not required, and differences among evaluators were less likely to occur. Therefore, the evaluation can easily be performed in older patients with non-nutrition-related occupations. However, some patients were susceptible to disease and edema owing to their body weight and were unsuitable for the study. Previous studies have shown an association between the GNRI and prognosis in patients with femoral fractures [20]. Most patients in this study had bone and joint diseases (42.5%), whereas fewer patients with cardiovascular and kidney diseases were prone to developing edema, suggesting that the GNRI strongly contributed to the prognosis, as reported in previous studies.

MNA^Ⓡ^-SF is also a nutritional screening tool specific for older adults, and its scores have been associated with prognosis [11, 21–23]. The results of this study showed an HR of 2.17 for malnutrition; however, no significant association was observed between risk, malnutrition, and prognosis. The MNA^Ⓡ^-SF questions were aimed at assessing weight loss, diet loss, and BMI status. Approximately half of the patients in this study had bone and joint diseases, and no significant difference was observed in the mean BMI between the survival and mortality groups. Therefore, the effect of the main disease on diet and body weight was relatively small and not significant based on the results of the MNA^Ⓡ^-SF evaluation.

There is little evidence showing the accuracy of the GLIM criteria in assessing the prognosis of older hospitalized patients, and only a few studies have evaluated the association between the GLIM criteria and patient’s prognosis [24]. In addition, the cut-off value for BMI that is used in the evaluation of phenotypic criteria remains under investigation [25]. A previous study conducted in older patients with diabetes showed that a high risk of malnutrition according to the GLIM criteria significantly contributed to the prediction of their prognosis at 8 years, but not in patients with moderate malnutrition [26], which is consistent with the results of this study. The GLIM criteria are the diagnostic standards for malnutrition, and their association with prognosis has been reported in various studies [27, 28]. In this study, (1) the assessment of phenotypic criteria, especially muscle mass, on all GLIM groups could not be measured using precision instruments; therefore, the diagnostic methods of the GLIM criteria could not be fully complied, and (2) the researchers may not have been able to fully perform the functions of the original GLIM criteria because the diagnosis was made based on the GLIM criteria for the first time.

In the GLIM criteria, the cut-off values for the three phenotypic criteria have not been determined, and the racial differences in BMI, muscle mass, and other factors, which are specific phenotypic criteria, have not been identified (adoption of reference values for small Asians). Hence, further studies are warranted.

This study has some limitations. First, this study was conducted in a single district hospital; therefore, its findings may not be applicable to all older patients owing to the main disease bias. Therefore, a multicenter study should be conducted. Second, the long-term prognosis is influenced by patients’ nutritional intake before discharge, but patient’s intake during hospitalization cannot be evaluated. Third, multivariate analysis was performed to identify the prognostic tools. However, none of the confounders could be adjusted for power loss or multicollinearity. Fourth, a plastic adipometer was used for obtaining anthropometric measurements. Similar kits were used in previous studies [29, 30], and a certain amount of evidence showed that the kits obtained accurate measurements. This kit was also used in this study; however, its accuracy and validity were not sufficient. Fifth, in patients who are unable to communicate about food intake, the adequacy of caregiver responses was not assessed.

## Conclusion

In conclusion, the GNRI was an independent predictor of prognosis 1 year after discharge in hospitalized older patients.

## Data Availability

The datasets generated and/or analyzed during the current study are not publicly available as there was no such approval for the study protocol. The data are available from the corresponding author upon reasonable request.

## Abbreviations

Alb: Albumin
BMI: Body mass index
CC: Calf circumference
CCI: Charlson Comorbidity Index
CI: Confidence interval
GLIM: Global leadership initiative on malnutrition
GNRI: Geriatric Nutritional Risk Index
HR: Hazard ratio
MNAⓇ-SF: Mini Nutritional Assessment-Short Form
TSF: Triceps skinfold thickness
